# Transient Hyperprolactinemia Associated With Semaglutide in a Patient With Hashimoto’s Thyroiditis

**DOI:** 10.1155/carm/3016596

**Published:** 2026-05-27

**Authors:** Gabriela N. F. Guimarães

**Affiliations:** ^1^ Independent Researcher, Porto Alegre, 91340-480, Brazil

**Keywords:** case report, GLP-1 receptor agonist, Hashimoto’s thyroiditis, hyperprolactinemia, semaglutide

## Abstract

Semaglutide, a glucagon‐like peptide‐1 (GLP‐1) receptor agonist, is widely used for weight management and glycemic control. While its metabolic and gastrointestinal effects are well characterized, its potential influence on pituitary function remains underexplored. To our knowledge, this appears to be among the first published reports of transient hyperprolactinemia associated with semaglutide use in a middle‐aged woman with well‐controlled Hashimoto’s thyroiditis. The patient developed prolactin levels approaching 100 ng/mL without clinical symptoms or MRI evidence of pituitary adenoma. Prolactin normalized after semaglutide discontinuation, without any pharmacologic intervention. This case raises the hypothesis that GLP‐1 receptor agonism may influence prolactin regulation in susceptible individuals, potentially through central neuroendocrine pathways involving prolactin‐releasing peptide (PrRP) signaling or dopaminergic modulation. Clinicians should consider medication‐related hyperprolactinemia in the differential diagnosis when elevated prolactin is detected during GLP‐1 receptor agonist therapy.

## 1. Introduction

Glucagon‐like peptide‐1 (GLP‐1) receptor agonists such as semaglutide are established treatments for Type 2 diabetes and have recently been approved for obesity management due to their potent weight‐reducing effects. In a pivotal trial of once‐weekly semaglutide (2.4 mg) for obesity, patients achieved an average weight loss of 15% of body weight over 68 weeks, making it a highly effective pharmacotherapy for weight reduction [1]. GLP‐1 analogs promote weight loss mainly by suppressing appetite and reducing caloric intake via central mechanisms, acting on GLP‐1 receptors in key brain regions that regulate feeding and reward [2]. Common adverse effects of GLP‐1 agonists include gastrointestinal symptoms (nausea, vomiting, and diarrhea), and there are known associations with thyroid C‐cell hyperplasia in rodents, but effects on pituitary function have not been well characterized. Hyperprolactinemia is typically caused by prolactin‐secreting pituitary adenomas or by medications (especially antipsychotics); primary hypothyroidism is another cause, via elevated thyrotropin‐releasing hormone (TRH) stimulating prolactin release [3]. To our knowledge, semaglutide‐associated hyperprolactinemia has not been well documented in humans. We describe a case of transient hyperprolactinemia in a patient with autoimmune hypothyroidism (Hashimoto’s disease) during treatment with low‐dose semaglutide for weight loss, which resolved after discontinuation of the GLP‐1 agonist.

## 2. Case Report

The patient is a 46‐year‐old woman with Hashimoto’s thyroiditis on stable levothyroxine replacement (75 μg daily). Her other medications included nightly melatonin (10 mg) for insomnia and a levonorgestrel‐releasing intrauterine system (LNG‐IUD; Mirena) for contraception. She was euthyroid on levothyroxine (baseline thyrotropin in 2020 was 1.6 mIU/L) and had no history of menstrual irregularities or galactorrhea; the menstrual pattern was not reliably assessable due to the LNG‐IUD. Detailed reproductive history prior to LNG‐IUD placement was not available in the retrospective record and is acknowledged as a limitation. Her body mass index (BMI) was 29.4 kg/m^2^ (weight: 80 kg and height: 1.65 m) in September 2020, and weights were recorded on a clinic scale at follow‐up visits. Semaglutide (Ozempic) was initiated for overweight with metabolic risk factors in the absence of diabetes, and the patient remained on 0.25 mg subcutaneously once weekly due to tolerability and clinical response. The dose was not escalated at the patient’s request, yet over the ensuing 8 months, she experienced significant weight loss of 19 kg (24% of initial weight), and by May 2021, her BMI had decreased to 22.5 kg/m^2^. The patient reported a nonrestrictive (approximately normocaloric to mildly hypercaloric) diet and no substantial change in physical activity. The magnitude of weight loss at a low dose is notable and may reflect individual sensitivity to GLP‐1 receptor agonism.

At the May 2021 follow‐up, hyperprolactinemia was detected incidentally on routine laboratory monitoring. Serum prolactin, measured by an electrochemiluminescence immunoassay, was 106 ng/mL (reference range: 5–25 ng/mL) and remained elevated on repeat sampling one week later (96 ng/mL), consistent with moderate hyperprolactinemia. Macroprolactin testing by polyethylene glycol precipitation was not performed. Around the time prolactin was first noted to be elevated, the patient denied acute intercurrent illness, recent major psychosocial stressors, or significant changes in sleep pattern beyond her longstanding baseline. Levothyroxine dose remained unchanged, and thyroid function tests were stable. She reported no recent initiation, discontinuation, or dose escalation of over‐the‐counter supplements or herbal products. She denied biotin supplementation. A targeted medication and supplement review confirmed no exposure to other common prolactin‐raising agents, including antipsychotics, metoclopramide/domperidone, opioids, systemic estrogens, verapamil, or antidepressant dose changes. The patient denied new symptoms; specifically, she had no galactorrhea, and any menstrual changes were difficult to discern due to the LNG‐IUD (she had intermittent spotting but no overt amenorrhea). There were no headaches, visual changes, or other neurological complaints. Physical exam was unremarkable, with no breast discharge on expression. Thyroid function tests at that time showed TSH 1.4 mIU/L with free T_4_ 1.2 ng/dL, indicating she remained biochemically euthyroid on her current levothyroxine dose. Other pituitary hormones were assessed to exclude concomitant pituitary dysfunction: morning cortisol was 14 μg/dL and insulin‐like growth factor‐1 was within age‐appropriate normal limits. Magnetic resonance imaging (MRI) of the sellar region with contrast was performed given the concern for a pituitary adenoma. Pituitary MRI with and without gadolinium contrast showed no sellar or suprasellar lesion and was interpreted by neuroradiology as normal.

With no structural lesion and no other secondary causes (e.g., no use of antipsychotic or dopamine‐blocking medications), a drug effect was considered. Semaglutide was the only recent addition to her regimen. In June 2021, semaglutide injections were discontinued to test the hypothesis that it was contributing to hyperprolactinemia. No dopamine agonists or other interventions were initiated. Three months after stopping semaglutide (September 2021), the patient’s prolactin levels had fallen to 24 and 35 ng/mL on two separate measurements, both within the near‐normal range. Her weight had stabilized around 64 kg (she regained only 3 kg after cessation of semaglutide). Thyroid labs remained in the euthyroid range on the same levothyroxine dose. Table 1 summarizes her key clinical and laboratory data before, during, and after semaglutide therapy.

**TABLE 1:** Key clinical and laboratory values before, during, and after semaglutide therapy in a 46‐year‐old woman with Hashimoto’s thyroiditis (normal reference ranges in parentheses).

Parameter	Baseline (September 2020)	On semaglutide (May 2021)	3 months after stopping (September 2021)
Body weight	80 kg	61 kg	64 kg
Body mass index	29.4 kg/m^2^	22.5 kg/m^2^	23.6 kg/m^2^
Prolactin	19 ng/mL^a^	106 ng/mL	24 ng/mL
TSH	1.6 mIU/L	1.4 mIU/L	2.0 mIU/L
Free T_4_	1.3 ng/dL	1.2 ng/dL	1.1 ng/dL
Estradiol	—	45 pg/mL^b^	—
FSH	—	12.4 mIU/mL^b^	—
MRI pituitary	—	No tumor (normal scan)	—

^a^Historical prolactin level measured in 2012 (while euthyroid and not on semaglutide).

^b^Random measurements during hyperprolactinemia; patient was perimenopausal on LNG‐IUD (not in a defined menstrual phase).

**FIGURE 1:**
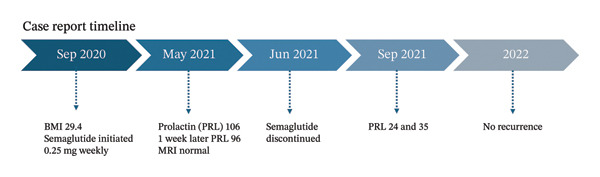
Case timeline showing the temporal association between semaglutide exposure and reversible hyperprolactinemia. Semaglutide 0.25 mg weekly was initiated in September 2020 (baseline BMI 29.4 kg/m^2^). In May 2021, serum prolactin (PRL) was elevated (106 ng/mL) and remained elevated on repeat testing one week later (96 ng/mL); pituitary MRI was normal. Semaglutide was discontinued in June 2021. By September 2021, PRL had normalized on two measurements (24 and 35 ng/mL), and no recurrence of hyperprolactinemia was observed on follow‐up through 2022.

The clinical course was consistent with possible semaglutide‐associated hyperprolactinemia although definitive causality could not be established in a single retrospective case. After semaglutide withdrawal, the patient’s prolactin returned to the normal or near‐normal range and she has had no evidence of recurrent clinically significant hyperprolactinemia on follow‐up testing through 2022 (Figure 1). Given the normalization of prolactin and lack of tumor on imaging, no dopamine agonist therapy was given. The patient continued thyroid hormone replacement for Hashimoto’s disease and has maintained a stable weight in the normal BMI range without GLP‐1 therapy.

## 3. Discussion

We report what appears to be a reversible hyperprolactinemia associated with semaglutide therapy. This case is unusual, as GLP‐1 receptor agonists are not commonly recognized to affect prolactin secretion. While causality cannot be established from a single case, the temporal association, exclusion of common alternative etiologies, negative pituitary imaging, and normalization after discontinuation are consistent with a possible medication‐related effect. Alternative causes of hyperprolactinemia were carefully considered. Medication effects: Aside from semaglutide, the patient’s only medications were levothyroxine (which would tend to lower prolactin if anything by restoring euthyroidism), melatonin, and an intrauterine progestin. Melatonin can transiently raise prolactin levels at night in some reports [3, 4], but our patient had been on long‐term melatonin without issues, making it an unlikely culprit. The levonorgestrel intrauterine device has mainly local hormone effects [5] and is not known to cause hyperprolactinemia [6]. Pelvic ultrasound was not obtained; therefore, ovarian morphology could not be evaluated as a potential confounder. Pituitary adenoma: Nonfunctioning pituitary microadenomas or “stalk effect” from pituitary stalk compression typically cause mild prolactin elevations (< 100 ng/mL) [7]. Conversely, prolactinomas usually cause higher levels, often > 100–200 ng/mL for microadenomas and > 250 ng/mL for macroadenomas [8]. Our patient’s prolactin (∼100 ng/mL) was in a gray zone that could be seen with a small prolactinoma, and indeed newer studies have noted that levels of 55–100 ng/mL can be associated with microadenomas in some cases. However, the lack of any lesion on high‐quality MRI and the full normalization of prolactin after drug withdrawal make a prolactinoma or other intrinsic pituitary pathology less likely. Primary hypothyroidism: Hypothyroidism is a known cause of hyperprolactinemia because elevated TRH in that state can costimulate lactotrophs; this is typically seen in untreated or severe hypothyroidism [9]. In this patient, TSH was normal on adequate levothyroxine therapy, so thyroid dysfunction was not a contributor. In summary, an idiosyncratic effect of semaglutide remained a plausible explanation. Macroprolactin (PEG precipitation) was not assessed, which is a limitation in moderate prolactin elevations; however, the reproducible measurements and normalization after discontinuation support a reversible drug‐associated effect. Sampling conditions (time of day, fasting status, and predraw rest) were not standardized or recorded in the retrospective chart; however, prolactin elevation was confirmed on repeat testing and later normalized following semaglutide discontinuation.

Important limitations remain. Macroprolactin testing was not performed, sampling conditions were not fully standardized, and gynecologic evaluation was incomplete, including the absence of pelvic ultrasound and limited interpretation of menstrual cyclicity because of the LNG‐IUD. In addition, substantial weight loss itself may have contributed to neuroendocrine adaptation. These factors preclude definitive causal attribution and support interpreting this case as hypothesis‐generating.

The biological basis for this association remains speculative. GLP‐1 receptor agonists act on central pathways involved in appetite, autonomic regulation, stress responses, and neuroendocrine signaling [10–13]. One hypothesis is that GLP‐1 signaling may interact with prolactin‐regulating circuits, including prolactin‐releasing peptide (PrRP) neurons and hypothalamic dopaminergic tone, thereby altering tonic inhibition of lactotrophs in susceptible individuals [14–18]. A related case involving dulaglutide described transient ovarian dysfunction with prolactin elevation, supporting biological plausibility but not establishing causality [19]. Conversely, controlled human data remain limited and inconsistent, including a trial in healthy men showing a slight reduction rather than an increase in prolactin during dulaglutide exposure [20]. Therefore, this proposed mechanism should be interpreted strictly as hypothesis generating.

The presence of Hashimoto’s thyroiditis in our patient likely did not play a direct role in the hyperprolactinemia, but it is worth discussing as a potential modulator. Autoimmune hypothyroidism, when untreated, is a recognized cause of mild hyperprolactinemia due to elevated TRH as mentioned above [3, 9]. In this case, however, the patient’s thyroid function was well controlled; her TSH was within the target range throughout, so there was no excess TRH drive. Could the autoimmune state itself predispose to hyperprolactinemia? Prolactin is known to have immunomodulatory properties and has been implicated in autoimmune diseases (for example, prolactin can enhance lymphocyte proliferation and is thought to contribute to systemic lupus erythematosus activity) [21]. Conversely, high prolactin levels can sometimes accompany autoimmune conditions as a secondary phenomenon [22, 23]. There is no evidence that Hashimoto’s thyroiditis by itself raises prolactin levels significantly, especially when the thyroid hormone levels are normalized. We, therefore, consider the thyroiditis to be a coincidental comorbidity rather than a contributor to this patient’s elevated prolactin. It remains important, though, in any patient with hyperprolactinemia to check thyroid function, as reconstitution of euthyroidism can itself correct prolactin elevations in cases of primary hypothyroidism.

Substantial weight loss represents an additional potential confounder. Caloric restriction, weight change, and physiologic stress may influence neuroendocrine hormones, including the gonadal and prolactin axes [24, 25]. Although the patient denied severe dietary restriction and prolactin normalized after semaglutide discontinuation, weight‐loss‐related neuroendocrine adaptation cannot be excluded as a partial contributor.

This case describes transient hyperprolactinemia temporally associated with low‐dose weekly semaglutide, with normalization after drug discontinuation and no pituitary lesion on MRI. Because this is a single retrospective case without macroprolactin testing, standardized sampling conditions, or complete gynecologic evaluation, causality cannot be established and alternative explanations, including neuroendocrine adaptations to substantial weight loss, cannot be excluded. Nevertheless, the temporal course raises the possibility that GLP‐1 receptor agonism may influence prolactin regulation in susceptible individuals. Clinicians encountering unexplained hyperprolactinemia in patients using GLP‐1 receptor agonists should review medications, confirm thyroid status, consider macroprolactin testing, and individualize decisions regarding continued therapy [26, 27].

## Funding

The author received no external funding for this work.

## Ethics Statement

This is a single anonymized case report from routine clinical practice in Brazil; formal ethics committee approval was not required. All potentially identifying information has been removed, and the report complies with the Declaration of Helsinki.

## Consent

Written informed consent was obtained from the patient for publication of the clinical details and any accompanying data.

## Conflicts of Interest

The author declares no conflicts of interest.

## Patient Perspective

Patient perspective was not obtained.

## Data Availability

The data supporting the findings of this case report are available from the corresponding author upon reasonable request.
